# Impact of conditioning intensity in T-replete haplo-identical stem cell transplantation for acute leukemia: a report from the acute leukemia working party of the EBMT

**DOI:** 10.1186/s13045-016-0248-3

**Published:** 2016-03-15

**Authors:** Marie T. Rubio, Bipin N. Savani, Myriam Labopin, Simona Piemontese, Emmanuelle Polge, Fabio Ciceri, Andrea Bacigalupo, William Arcese, Yener Koc, Dietrich Beelen, Zafer Gülbas, Depei Wu, Stella Santarone, Johanna Tischer, Boris Afanasyev, Christoph Schmid, Sebastian Giebel, Mohamad Mohty, Arnon Nagler

**Affiliations:** Service d’Hématologie et de Thérapie cellulaire, Saint Antoine Hospital, Paris, France; INSERM UMR 938, Paris, France; Université Pierre et Marie Curie, Paris, France; Acute Leukemia Working Party of EBMT, Paris, France; Vanderbilt University Medical Center, Nashville, TN USA; EBMT Paris study office/CEREST-TC, Paris, France; Ospedale San Raffaele s.r.l., Ematologia, Milan, Italy; Department of Haematology II, Ospedale San Martino, Genoa, Italy; Rome Transplant Network, Stem Cell Transplant Unit, Tor Vergata University, Rome, Italy; Stem Cell Transplant Unit, Medical Park Hospitals, Antalya, Turkey; Department of Bone Marrow Transplantation, University Hospital, Essen, Germany; Bone Marrow Transplantation Department, Anadolu Medical Center Hospital, Kocaeli, Turkey; Department of Hematology, First Affiliated Hospital of Soochow University, Suzhou, China; Ospedale Civile BMT Center, Pescara, Italy; Department of Internal Medicine III, Ludwig-Maximilians-University Hospital of Munich-Grosshadern, Munich, Germany; SPb State I. Pavlov Medical University, St. Petersburg, Russia; Klinikum Augsburg, University of Munich, Munich, Germany; Department of Bone Marrow Transplantation and Hemato-Oncology, Cancer Center, Gliwice, Poland; Hematology Division, Chaim Sheba Medical Center, Tel Hashomer, Israel

**Keywords:** Allogeneic stem cell transplantation, Haplo-identical donor, Conditioning regimen, Acute Leukemia, Toxicity, Anti-leukemic effect

## Abstract

**Background:**

Increasing numbers of patients are receiving haplo-identical stem cell transplantation (haplo-SCT) for treatment of acute leukemia with reduced intensity (RIC) or myeloablative (MAC) conditioning regimens. The impact of conditioning intensity in haplo-SCT is unknown.

**Methods:**

We performed a retrospective registry-based study comparing outcomes after T-replete haplo-SCT for patients with acute myeloid (AML) or lymphoid leukemia (ALL) after RIC (*n* = 271) and MAC (*n* = 425). Regimens were classified as MAC or RIC based on published criteria.

**Results:**

A combination of post-transplant cyclophosphamide (PT-Cy) with one calcineurin inhibitor and mycophenolate mofetil (PT-Cy-based regimen) for graft-versus-host disease (GVHD) prophylaxis was used in 66 (25 %) patients in RIC and 125 (32 %) in MAC groups. Patients of RIC group were older and had been transplanted more recently and more frequently for AML with active disease at transplant. Percentage of engraftment (90 vs. 92 %; *p* = 0.58) and day 100 grade II to IV acute GVHD (24 vs. 29 %, *p* = 0.23) were not different between RIC and MAC groups. Multivariable analyses, run separately in AML and ALL, showed a trend toward higher relapse incidence with RIC in comparison to MAC in AML (hazard ratio (HR) 1.34, *p* = 0.09), and no difference in both AML and ALL in terms of non-relapse mortality (NRM) chronic GVHD and leukemia-free survival. There was no impact of conditioning regimen intensity in overall survival (OS) in AML (HR = 0.97, *p* = 0.79) but a trend for worse OS with RIC in ALL (HR = 1.44, *p* = 0.10). The main factor impacting outcomes was disease status at transplantation (HR ≥ 1.4, *p* ≤ 0.01). GVHD prophylaxis with PT-Cy-based regimen was independently associated with reduced NRM (HR 0.63, *p* = 0.02) without impact on relapse incidence (HR 0.99, *p* = 0.94).

**Conclusions:**

These data suggest that T-replete haplo-SCT with both RIC and MAC, in particular associated with PT-Cy, are valid options in first line treatment of high risk AML or ALL.

**Electronic supplementary material:**

The online version of this article (doi:10.1186/s13045-016-0248-3) contains supplementary material, which is available to authorized users.

## Background

Haplo-identical hematopoietic stem cell transplantation (haplo-SCT) is an attractive transplant procedure since it provides a possibility of transplantation to almost all patients needing an allogeneic SCT. Historical approaches of haplo-SCT performed with unmanipulated bone marrow stem cell grafts, standard myeloablative conditioning, and graft-versus-host disease (GVHD) prophylaxis were associated with high risks of graft rejection and severe GVHD due to uncontrolled bi-directional recipient and donor allo-reactivity [1–4]. Although the development of T cell depleted haplo-SCT has allowed to control the risk of severe GVHD, the poor T cell immune reconstitution observed in these patients is associated with a high incidence of life-threatening infections [5–10]. In the last decade, several strategies of T cell replete bone marrow (BM) or peripheral blood (PB) haplo-SCT have been developed with both reduced intensity (RIC) and fully intensive myeloablative conditioning (MAC) regimens and variable GVHD prophylaxis strategies [11–26]. While some groups have used a combination of anti-thymocyte globulin (ATG) with immunosuppressive agents with or without monoclonal antibodies such as rituximab and basiluximab [11–16], others have adopted the Baltimore’s strategy [17–26] of in vivo T cell depletion by the administration of high doses of cyclophosphamide (Cy) on days 3 and 4 post-SCT (PT-Cy). These approaches have improved immune reconstitution and reduced non-relapse mortality (NRM) of haplo-SCT [20, 27–29]. In single center experiences, these new haplo-SCT platforms allow similar transplant outcomes than allo-SCT performed with HLA-matched donors [30–37]. However, the optimal conditioning regimen for haplo-SCT in acute leukemia remains a question of debate.

In HLA-matched related or unrelated allogeneic SCT for acute leukemia, several studies have reported a dose-dependent effect of the intensity of the conditioning regimen on disease control [38–40]. In this context, the reduced risk of relapse after MAC is counterbalanced by higher NRM leading to similar overall survival in MAC and RIC [41–48]. In T-replete haplo-SCT, low risks of acute and chronic GVHD, resulting in <20 % NRM at 1 to 5 years after RIC and MAC, have been reported [17–26]. Relapse incidence, however, varies from 35 to 60 % at 1-year post-transplant and remained the major event after haplo-SCT performed with RIC and PT-Cy [17–19, 23, 26]. Although these differences could be related to disease risk at transplant [24, 25, 49], these observations raise the question of the role of the conditioning regimen intensity on leukemic control and transplant outcomes after T-replete haplo-SCT.

To address this question, we performed a large retrospective registry study to compare the transplant outcomes of 696 patients receiving a haplo-SCT after RIC (*n* = 271) to those transplanted after MAC (*n* = 425) for acute leukemia.

## Results

### Patient, disease, and transplant characteristics

Details of patients, disease, and transplant characteristics are summarized in Table 1. Six hundred and ninety-six patients with AL were included in the study. Two hundred and seventy-one patients received RIC and 425 MAC regimen before haplo-SCT between 2001 and 2012. Patients of the RIC group were older with median age of 53 years (range, 18–76) in comparison to 38 years (range, 18–4) for the MAC group (*p* < 0.0001). Only 45 % of the patients were ≤50 years of age in the RIC group vs. 70 % in the MAC group (*p* < 0.0001). The median follow-up of surviving patients in the RIC group was 15 (range, 1–73) months, while that of the MAC group was 22 (range, 1–142) months (*p* = 0.007). Significantly higher numbers of patients were transplanted for acute myeloid (AML) in the RIC than in the MAC group (80 vs. 67 %), while more lymphoid leukemia (ALL) recipients were documented in the MAC cohort (33 vs. 20 %; *p* < 0.0001). There were more patients in CR1 in the MAC group (41 vs. 29 %) and with active disease in RIC compared to MAC groups (47 vs. 31 %; *p* < 0.0001). The other pre-transplant characteristics were similar between RIC and MAC groups, and the majority of patients were transplanted from 2008 to 2012 in both groups (91.5 % in RIC and 82.6 % in MAC) (Table 1).

**Table 1:** Patient and disease characteristics

Patient characteristics	RIC	MAC	*p* value
Number of patients	271	425	
Recipient age at SCT (years, range)	53 (18–76)	38 (18–74)	<10^−4^
Recipient age (by classes), *n* (%)			<0.0001
< 30	46 (17.0 %)	148 (34.8 %)	
30–40	32 (11.8 %)	82 (19.3 %)	
40–50	45 (16.6 %)	98 (23.1 %)	
≥ 50	148 (54.6 %)	97 (22.8 %)	
Recipient gender, *n* (%)			0.42
Male	153 (56.5 %)	252 (59.6 %)	
Female	118 (43.5 %)	171 (40.4 %)	
Year of SCT (median), year (%)	2010	2011	0.65
2001–2007	23 (8.5 %)	74 (17.4 %)	<0.0001
2008–2012	248 (91.5 %)	351 (82.6 %)	
Interval from diagnosis to SCT (days, range)	299 (52–3892)	272 (41–3689)	0.24
Donor age	37 years (19–71)	42 years (12–70)	0.83
Donor age (by classes), year (%)			0.55
< 30	39 (29.3 %)	54 (29.2 %)	
30–40	32 (24.1 %)	33 (17.8 %)	
40–50	29 (21.8 %)	46 (24.9 %)	
≥ 50	33 (24.8 %)	52 (28.1 %)	
Donor gender, *n* (%)			0.69
Male	138 (50.9 %)	223 (52.5 %)	
Female	133 (49.1 %)	202 (47.5 %)	
Female donor to male recipient, *n* (%)	76 (28 %)	119 (28.1 %)	0.98
Diagnosis, *n* (%)			<0.0001
AML	217 (80.1 %)	286 (67.3 %)	
Good cytogenetics	8 (6 %)	19 (14 %)	
Intermediate cytogenetics	47 (37 %)	53 (39 %)	
Poor cytogenetics	19 (15 %)	18 (13 %)	
Secondary AML	54 (42 %)	45 (33 %)	
Missing cytogenetics	89 (41 %)	151 (53 %)	
ALL	54 (19.9 %)	139 (32.7 %)	
Phi+ ALL	15 (54 %)	27 (36 %)	
Missing cytogenetics	26 (48 %)	64 (46 %)	
Disease status at SCT, *n* (%)			<0.0001
CR1	78 (28.8 %)	175 (41.2 %)	
≥ CR2	67 (24.7 %)	118 (27.8 %)	
Active disease	126 (46.5 %)	132 (31.1 %)	
Karnosky at SCT, *n* (%)			0.05
≤ 80 %	101 (39.9 %)	109 (32 %)	
> 80 %	152 (60.1 %)	232 (68 %)	
Missing	18 (6.7 %)	84 (19.8 %)	
Patient positive CMV serology, *n* (%)	213 (79.8 %)	311 (78.3 %)	0.66
CMV risk, *n* (%)			0.06
Low	35 (13.3 %)	59 (15.2 %)	
Intermediate	169 (64.2 %)	272 (69.3 %)	
High	59 (22.4 %)	56 (14.5 %)	

*AML* acute myeloid leukemia, *ALL* acute lymphoid leukemia, *CMV* cytomegalovirus, *CMV risk* low = negative recipient and donor serology, *high* positive recipient and negative donor serology, *intermediate* all other combinations, *CR* complete remission, *SCT* stem cell transplantation

The indication for haplo-SCT was high risk disease in the majority of patients in both groups, with 71 and 59 % of patients in RIC and MAC groups, respectively, transplanted in advanced phase or active disease.

Details of transplant characteristics, conditioning, and GVHD prophylaxis regimens are summarized in Table 2. The majority of patients in the RIC group received PB stem cells (65 %) compared to a similar distribution of PB and BM as stem cell source in the MAC cohort (52.5 and 47.5 %, respectively; *p* = 0.001). The percentage of patients receiving in vivo T cell depletion, mainly performed with Thymoglobulin, was not significantly different between the two groups (*p* = 0.26). Apart from in vivo T cell depletion, GVHD prophylaxis consisted of the combination of one calcineurin inhibitor with mycophenolate mofetyl (MMF) alone, or in association with methotrexate (MTX) +/− Basiluximab (anti-CD25) or post-transplant cyclophosphamide (PT-Cy). A calcineurin inhibitor + MMF and/or MTX +/− Basimuximab was used in 31 and 54 % of the patients in the RIC and MAC groups, respectively, while the association of a calcineurin inhibitor + MMF with PT-Cy (PT-Cy + CsA/Tacro + MMF) was applied in 25 and 32 % of patients of the RIC and MAC groups, respectively. Another frequent combination was sirolimus and MMF, used in 33 % of patients of the RIC group (Table 2). The choice of conditioning and GVHD prophylaxis was dependent on centers’ protocols and strategies of transplantation.

**Table 2:** Transplant characteristics

Transplant characteristics	RIC	MAC	*p* value
Number of patients	271	425	
Source of stem cells			0.001
BM	95 (35.1 %)	202 (47.5 %)	
PB	176 (64.9 %)	223 (52.5 %)	
Conditioning regimen			
Chemotherapy-based	200 (73.8 %)	306 (72 %)	
TBI-based	71 (26.2 %)	119 (28 %)	
In vivo T depletion			0.26
Yes	140 (51.7 %)	201 (47.3 %)	
Thymoglobulin	127 (91 %)	185 (92 %)	
Lymphoglobulin	2 (1 %)	1 (0.5 %)	
Alemtuzumab	11 (8 %)	15 (7.5 %)	
No	131 (48.3 %)	224 (52.7 %)	
Post-transplant GVHD prophylaxis			<0.0001
CsA/FK506 + MMF or MTX	61 (23.2 %)	120 (30.3 %)	
CsA + MMF + MTX	2 (0.8 %)	55 (13.9 %)	
CsA + MMF + MTX + Basiliximab	19 (7.2 %)	40 (10.1 %)	
PT-Cy + CsA/FK506 + MMF	66 (25.1 %)	125 (31.6 %)	
Sirolimus + MMF	87 (33.1 %)	10 (2.5 %)	
Other	28 (10.6 %)	46 (11.6 %)	
DLI			NS
No DLI	247 (91.1 %)	389 (92.5 %)	
Pre-emptive DLI	11 (4.1 %)	14 (3.3 %)	
DLI after relapse	13 (4.8 %)	22 (5.2 %)	

*BM* bone marrow, *Bu* busulfan, *CSA* cyclosporine, *Cy* cyclophosphamide, *DLI* donor lymphocyte injection, *Flu* fludarabine, *MMF* mycophenolate mofetyl, *MTX* methotrexate, *PB* peripheral blood, *PT-CY* post-transplant cyclophosphamide, *TBI* total body irradiation

Donor lymphocyte infusion (DLI) was reported in 24 (8.9 %) in RIC and 36 (8.5 %) in MAC groups. Among those, DLI was pre-emptive for 11 patients (4 % of patients) in RIC and 14 (3 %) in MAC groups.

### Engraftment and GVHD

Conditioning regimen specific engraftment and GVHD are summarized in Table 3. Ninety percent of patients in the RIC group engrafted vs. 92 % in the MAC group (*p* = 0.58). The median day for ANC > 500/μL was 17 (range, 3–75) and 18 (range, 6–63) days in MAC and RIC groups, respectively (*p* = 0.007). The incidences of day 100 grade II–IV (29 vs. 24 %; *p* = 0.23) and III–IV (10.7 vs. 10.9 %; *p* = 0.96) acute GVHD were not significantly different between MAC and RIC groups, respectively. As shown in Table 5, in multivariate analysis, the only factor associated with increased grade II–IV acute GVHD was the use of PB in comparison to BM stem cells (hazard ratio (HR) 1.96; 95 % CI, 1.32–2.92; *p* = 0.0008). Two-year incidence of chronic GVHD was similar between the different conditioning groups: 25 % (95 % CI, 19–31) in RIC vs. 32 % (95 % CI, 28–37) in MAC groups; (*p* = 0.14). There was no difference of incidence of cGVHD in AML and ALL groups (Table 4). In multivariate analysis, chronic GVHD was not significantly different between RIC and MAC groups (HR 0.80; 95 % CI, 0.56–1.14; *p* = 0.21). The only factor associated with chronic GVHD was the use of PB in comparison to BM stem cells (HR 1.64; 95 % CI, 1.16–2.30; *p* = 0.005) (Table 5).

**Table 3:** Engraftment and GVHD

	RIC	MAC	*p* value
Total number of patients	271	425	
Engraftment	235 (90.4 %)	383 (91.7 %)	0.58
Non-engraftment	25 (9.8 %)	35 (8.4 %)	
Missing	11	7	
Acute GVHD			
Grade 0–I, *n* (%)	202 (75.6 %)	293 (71.5 %)	0.23
Grade II–IV, *n* (%)	65 (24.4 %)	117 (28.5 %)	
Grade III–IV, *n* (%)	29 (10.9 %)	45 (10.7 %)	0.96
Missing, *n*	4	15	
Chronic GVHD^a^	24.7 % (19–30.9)	32.1 % (27.3–37.1)	0.14
Limited, *n*	29	76	
Extensive, *n*	22	36	
Missing, *n*	2	5	

*GVHD* graft-versus-host disease

^a^2-year cumulative incidence

**Table 4:** Comparison of 2-year outcomes between MAC and RIC haplo-SCT according to disease status

Disease	Patients group and *p* value	RI	NRM	LFS	OS	cGVHD
Diagnosis	AML	36.1 % (31.6–40.6)	31.6 % (27.4–35.9)	32.2 % (27.7–36.7)	37.7 % (33.1–42.4)	29.2 % (24.8–33.7)
	ALL	40 % (32.5–47.3)	32.9 % (28.6–37.2)	27.2 % (20.2–34.1)	33.3 % (25.9–40.7)	29.8 % (22.8–37.2)
	p	0.43	0.87	0.45	0.40	0.67
All patients						
CR1	RIC	30.1 % (19.1–41.9)	31.1 % (24.1–38.3)	38.9 % (26.4–51.3)	46.9 % (34.1–59.8)	26 % (15–38.3)
	MAC	21.5 % (15.4–28.3)	29.1 % (22.2–36.3)	49.1 % (41.2–57.1)	55.4 % (47.5–63.4)	41 % (32.8–48.9)
	p	0.21	0.80	0.42	0.64	0.10
≥ CR2	RIC	38.5 % (25.8–51.1)	36.8 % (28.2–45.5)	24.6 % (13.1–36.1)	29.8 % (17.7–41.9)	29.6 % (17.5–42.8)
	MAC	30 % (21.5–39)	27.8 % (19.7–36.4)	42.2 % (32.6–51.7)	47.7 % (38–57.5)	37 % (27.4–46.6)
	p	0.38	0.25	0.06	0.07	0.53
Active dis.	RIC	55.8 % (45.9–64.6)	32.9 % (24.5–41.5)	11.3 % (5–17.5)	14.3 % (7.3–21.3)	21.4 % (13.8–30.1)
	MAC	51.9 % (42.3–60.6)	37.1 % (28.6–45.7)	11 % (4.7–17.2)	18.5 % (11.2–25.7)	15.2 % (9.2–22.7)
	P	0.52	0.38	0.64	0.60	0.29

*Active dis* active disease, *cGVHD* chronic graft-versus-host disease, *CR* complete remission, *LFS* leukemia-free survival, *NRM* non-relapse mortality, *MAC* myeloablative conditioning, *OS* overall survival, *RI* relapse incidence, *RIC* reduced intensity conditioning

**Table 5:** Multivariate analysis

	Relapse		NRM		Acute GVHD		Chronic GVHD		LFS		OS	
	*p* value	HR (95 % CI)	*p* value	HR (95 % CI)	*p* value	OR (95 % CI)	*p* value	HR (95 % CI)	*p* value	HR (95 % CI)	*p* value	HR (95 % CI)
RIC vs. MAC												
All	0.07	1.31 (0.98–1.75)	0.76	0.95 (0.69–1.31)	0.32	0.82 (0.55–1.21)	0.21	0.80 (0.56–1.14)	0.27	1.13 (0.91–1.40)	0.44	1.09 (0.87–1.36)
AML	0.09	1.34 (0.95–1.87)	0.17	0.78 (0.54–1.12)					0.74	1.04 (0.81–1.34)	0.79	0.97 (0.75–1.25)
ALL	0.5	1.23 (0.67–2.26)	0.18	1.5 (0.83–2.72)					0.17	1.34 (0.88–2.05)	0.10	1.44 (0.94–2.22)
Age at SCT												
1. <30 (ref)		1.00		1.00		1.00		1.00		1.00		1.00
2. (30–40) vs. 1	0.91	1.02 (0.68–1.54)	0.27	0.79 (0.51–1.20)	0.98	1.01 (0.60–1.70)	0.25	0.75 (0.46–1.22)	0.47	0.90 (0.67–1.20)	0.52	0.91 (0.67–1.23)
3. (40–50) vs. 2	0.43	0.84 (0.55–1.28)	0.74	1.08 (0.69–1.69)	0.98	1.01 (0.58–1.77)	0.70	0.91 (0.56–1.48)	0.74	0.95 (0.70–1.29)	0.79	0.96 (0.69–1.32)
4. ≥ 50 vs. 3	0.11	1.33 (0.93–1.90)	0.48	0.87 (0.59–1.28)	0.06	1.62 (0.98–2.70)	0.44	1.18 (0.77–1.79)	0.52	1.09 (0.84–1.42)	0.79	1.04 (0.79–1.36)
ALL vs. AML	*0.04*	*1.39 (1.02*–*1.91)*	0.37	1.16 (0.84–1.61)	0.65	1.10 (0.73–1.66)	0.50	1.13 (0.79–1.63)	*0.03*	*1.28 (1.02*–*1.61)*	*0.03*	*1.30 (1.03*–*1.64)*
Status at SCT												
CR1 (ref)		1.00		1.00		1.00		1.00		1.00		1.00
≥ CR2 vs. CR1	*0.05*	*1.46 (1.01*–*2.13)*	0.45	1.15 (0.80–1.63)	0.35	1.24 (0.80–1.92)	0.23	1.25 (0.87–1.78)	*0.05*	*1.30 (1.00*–*1.67)*	*0.01*	*1.40 (1.07*–*1.83)*
Active dis. vs. CR1	*<10*–*4*	*4.43 (3.20*–*6.12)*	*0.0002*	*1.86 (1.34*–*2.59)*	0.85	1.04 (0.68–1.60)	0.45	1.17 (0.78–1.75)	*<10*–*4*	*2.94 (2.34*–*3.70)*	*<10*–*4*	*2.92 (2.30*–*3.72)*
PB vs. BM	0.85	1.03 (0.77–1.37)	0.59	1.09 (0.80–1.48)	*0.0008*	1.96 (1.32–2.92)	*0.005*	*1.64 (1.16*–*2.30)*	0.63	1.05 (0.85–1.30)	0.58	1.06 (0.85–1.32)
PT-Cy-based vs. other	0.94	0.99 (0.72–1.36)	*0.02*	*0.63 (0.44*–*0.92)*	0.11	0.69 (0.44–1.09)	0.46	1.15 (0.79–1.69)	0.10	0.82 (0.64–1.04)	0.16	0.83 (0.65–1.07)
Year of SCT												
2001–2005 (ref)		1.00		1.00		1.00		1.00		1.00		1.00
2005–2007	0.86	1.08 (0.48–2.43)	0.74	0.89 (0.45–1.77)	0.62	1.24 (0.44–3.51)	0.17	2.03 (0.74–5.56)	0.89	0.96 (0.57–1.63)	0.84	1.06 (0.61–1.83)
2008–2012	0.98	0.99 (0.47–2.09)	0.22	0.68 (0.36–1.27)	0.27	1.68 (0.67–4.22)	0.14	2.04 (0.80–5.19)	0.40	0.81 (0.50–1.31)	0.60	0.87 (0.53–1.44)

*Active dis* active disease, *BM* bone marrow, *PT-Cy-based* post-transplant cyclophosphamide associated to one calcineurin inhibitor and mycophenolate mofetyl, *CR* complete remission, GVHD graft-versus-host-disease, *LFS* leukemia-free survival, *NRM* non-relapse mortality, *MAC* myeloablative conditioning, *OS* overall survival, *PB* peripheral blood, *Ref* reference, *RIC* reduced intensity conditioning, *SCT* allogeneic stem cell transplantation

### Toxicity and NRM

There was no difference in NRM at 2 years between RIC and MAC groups in univariate (33 %; 95 % CI, 29–38; after RIC vs. 31 %; 95 % CI, 27–36 after MAC; *p* = 0.88) and multivariate analyses (HR 0.95; 95 % CI, 0.69–1.31; *p* = 0.76) (Table 5) in the total population of patients. When analyzed separately, there was no difference neither between AML and ALL in univariate analysis (31.6 %; 95 % CI, 27–36; in AML vs. 33 %; 95 % CI, 28–37 in ALL; *p* = 0.87) (Table 4). In addition, in multivariate analysis, the intensity of the conditioning had no significant impact on NRM in AML and ALL groups (Table 5).

**Fig. 1:**
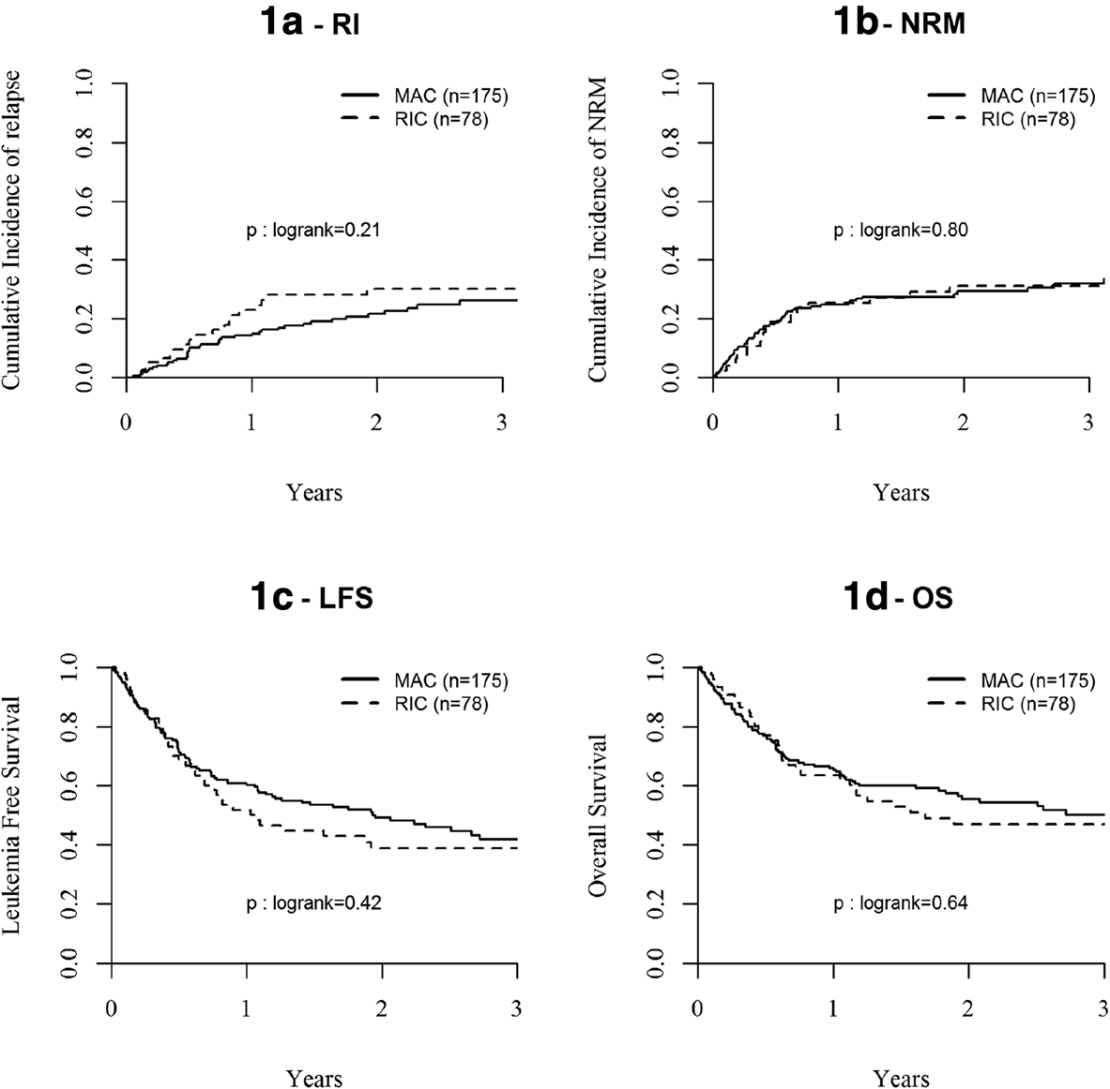
Probability of (*1a*) relapse incidence (RI); (*1b*) non-relapse mortality (NRM); (*1c*) leukemia-free survival; and (*1d*) overall survival (OS) after MAC or RIC haplo-SCT for AL in CR1

Among all patients, in univariate analysis, there was no difference in NRM at 2 years between the two groups for patients transplanted in CR1 (Table 4 and Fig. 1), in ≥CR2 or with active disease (Table 4). In multivariate analysis, however, active disease at transplant was associated with a higher risk of NRM (HR 1.86; 95 % CI, 1.34–2.59; *p* = 0.0002), while the use of PT-Cy + CsA/Tacro + MMF was associated with decreased NRM as compared to other GVHD prophylaxis (HR 0.63; 95 % CI, 0.44–0.92; *p* = 0.02) (Table 5 and Fig. 3). There was no impact of recipient age at transplant, type of disease (ALL vs. AML), source of stem cells (PB vs. BM), and year of transplant (Table 5).

The main causes of NRM were infectious complications in 53 (32 %) vs. 75 (33 %) patients and GVHD in 23 (14 %) vs. 36 (16 %) of patients in RIC vs. MAC groups, respectively. Death from organ toxicity was very low in both groups. In particular, sinusoidal obstructive syndrome (SOS) was reported in 2 (1.2 %) and 5 (2.2 %) of patients in RIC and MAC groups, respectively.

### Relapse

**Fig. 2:**
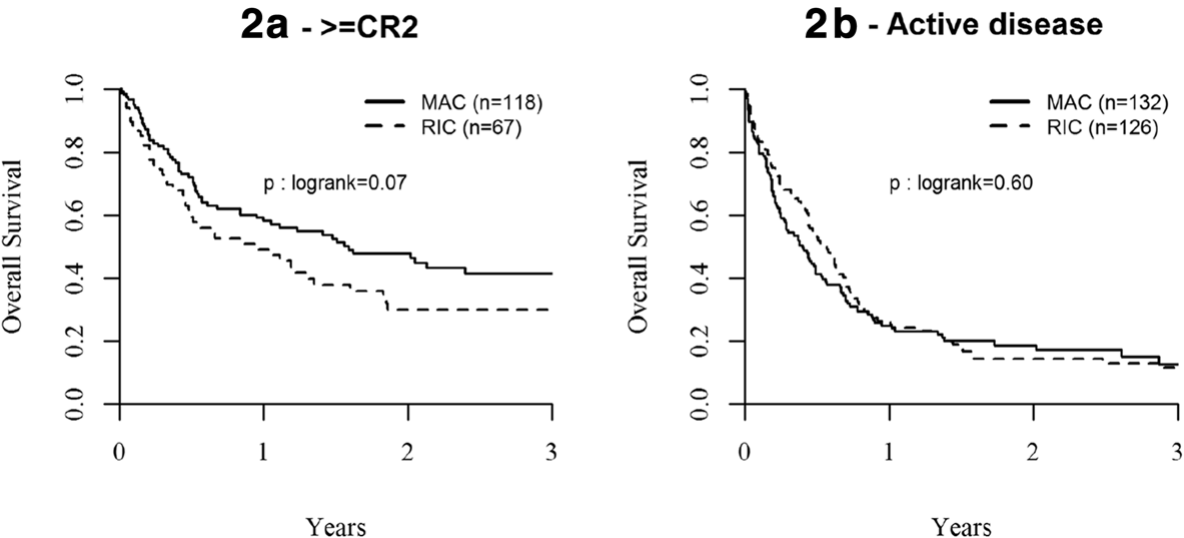
Probability of overall survival (OS) after MAC or RIC haplo-SCT for AL in (*2a*) ≥ CR2 and (*2b*) with active disease at transplant

Overall RI at 2 years was comparable between AML and ALL (36.1 % in AML and 40 % in ALL, *p* = 0.43) (Table 4). However, since disease status at transplant was significantly different between the two conditioning groups (Table 1), relapse incidence and survivals were analyzed separately for all patients transplanted in CR1, ≥ CR2, and with active disease (Table 4). Overall RI at 2 years for patients transplanted in CR1, ≥ CR2, and with active disease were 23.9, 33.1, and 53.6 %, respectively. In univariate analysis, RI at 2 years was similar between RIC and MAC groups for patients in CR1 (30 %; 95 % CI, 19–42 vs. 22 %; 95 % CI, 14–28 %; respectively; *p* = 0.21) (Table 4 and Fig. 1), in ≥CR2 (39 %; 95 % CI, 26–51 vs. 30 %; 95 % CI, 22–39 %; respectively; *p* = 0.38) or with active disease (56 %; 95 % CI, 47–65 vs. 52 %; 95 % CI, 42–61 %; respectively; *p* = 0.52) (Table 4). In multivariate analysis, there was a trend to a slight increase of RI in the RIC vs. MAC group (HR 1.31; 95 % CI, 0.98–1.75; *p* = 0.07) (Table 5). This trend was particularly observed in AML patients (HR 1.34; 95 % CI, 0.95–1.87; *p* = 0.09) (Table 5). In addition, the risk of relapse was significantly higher for patients transplanted in ≥CR2 (HR 1.46; 95 % CI, 1.01–2.13; *p* = 0.05) or with active disease (HR 4.43; 95 % CI, 3.20–6.12; *p* < 0.0001) compared to CR1, and in ALL compared to AML (HR 1.39; 95 % CI, 1.02–1.91; *p* = 0.04) (Table 5). Despite reduced NRM, the RI was similar in patients having received PT-Cy + CsA/Tacro + MMF in comparison to other GVHD prophylaxis (HR 0.99; 95 % CI, 0.72–1.36; *p* = 0.94) (Table 5 and Fig. 2).

### Leukemia-free survival

LFS at 2 years was comparable between AML and ALL (32.2 % in AML and 27.2 % in ALL, *p* = 0.67) (Table 4). Overall LFS at 2 years for all patients transplanted in CR1, ≥CR2, and with active disease were 46.3, 35.7, and 11.4 %, respectively. LFS at 2 years was similar between RIC and MAC groups for patients transplanted in CR1 (39 %; 95 % CI, 26–51 vs. 49 %; 95 % CI, 41–57 %, respectively, *p* = 0.42) (Table 4 and Fig. 1) or with active disease (11 %; 95 % CI, 5–18 vs. 11 %; 95 % CI, 5–17 %, respectively, *p* = 0.64) (Table 4). There was a trend towards worse LFS in RIC vs. MAC groups for patients transplanted in ≥CR2 (25 %; 95 % CI, 13–36 vs. 42 %; 95 % CI, 33–52 %; respectively; *p* = 0.06) (Table 4). However, in multivariate analysis, LFS was no different between RIC and MAC groups for all patients (HR 1.13; 95 % CI, 0.91–1.40, *p* = 0.27), as well as for AML (HR 1.04; 95 % CI, 0.81–1.34, *p* = 0.74) and ALL (HR 1.34; 95 % CI, 0.88–2.05, *p* = 0.17) patients (Table 5). Multivariate analysis showed lower LFS in patients transplanted in ≥CR2 (HR 1.30; 95 % CI, 1.00–1.67; *p* = 0.05) or with active disease (HR 2.94; 95 % CI, 2.34–3.70; *p* < 0.0001) compared to CR1, and in ALL compared to AML (HR 1.28; 95 % CI, 1.02–1.61; *p* = 0.03) (Table 5).

### Overall survival

Overall survival (OS) at 2 years was comparable between AML and ALL (37.7 % in AML and 33.3 % in ALL, *p* = 0.40) (Table 4). Overall OS at 2 years for patients transplanted in CR1, ≥CR2, and with active disease were 53.2, 41, and 16.3 %, respectively. OS at 2 years was similar in RIC and MAC groups for patients transplanted in CR1 (47 %; 95 % CI, 34–60 vs. 55 %; 95 % CI, 48–63 %, respectively, *p* = 0.64) (Table 4 and Fig. 1) or with active disease (14 %; 95 % CI, 7–21 vs. 19 %; 95 % CI, 11–26 %, respectively, *p* = 0.60) (Table 4 and Fig. 2). There was a trend towards worse OS in RIC as compared to MAC groups for patients transplanted in ≥CR2 (30 %; 95 % CI, 18–42 vs. 48 %; 95 % CI, 38–58 %; respectively; *p* = 0.07) (Table 4 and Fig. 2). In multivariate analysis, OS was no different between RIC and MAC groups in the total cohort of patients (HR 1.09; 95 % CI, 0.87–1.36, *p* = 0.44) (Table 5) and in AML patients (HR 0.97; 95 % CI, 0.75–1.25, *p* = 0.79). Although not significant, there was a trend for a worse OS with RIC in ALL patients (HR 1.44; 95 % CI, 0.94–2.22, *p* = 0.10) (Table 5). Of note, 39 % of ALL patients receiving a RIC had active disease at transplantation in comparison to 24 % of those transplanted with MAC (*p* = 0.03). Multivariate analysis also showed lower OS in patients transplanted in ≥CR2 (HR 1.40; 95 % CI, 1.07–1.83; *p* = 0.01) or with active disease (HR 2.92; 95 % CI, 2.30–3.72; *p* < 0.0001) compared to CR1, and in ALL compared to AML (HR 1.30; 95 % CI, 1.03–1.64; *p* = 0.03) (Table 5).

## Discussion

The development of T-replete haplo-SCT for the treatment of hematological malignancies has been considerable over the last 5 to 10 years. Several RIC and MAC regimens have been designed by different groups at the same time with GVHD prophylaxis regimens based either on the Baltimore’s approach using PT-Cy [17–26] or the combination of several immunosuppressive drugs with in vivo T cell depletion with monoclonal antibodies [11–16]. They all demonstrated the feasibility of such transplants with limited NRM (≤20 % at 1 year) and promising survival rates, in particular in patients transplanted in CR [24, 25, 49]. However, comparative studies between the different transplant strategies had not been performed. Our study represents the first large retrospective analysis comparing transplant outcomes between RIC and MAC T-replete haplo-SCT for AML and ALL. In AML, our results show similar LFS and OS in patients receiving RIC and MAC regimens after adjustment for disease risk at transplant. In ALL, there was a trend for worse OS with RIC not related to increase relapse but rather a trend for increase NRM. This might be explained by higher proportions of patients with advanced disease in the RIC group.

In multivariate analysis, two factors impacted LFS and OS: disease status at transplant in all patients and in the AML and ALL subgroups (data not shown), and type of leukemia. Patients transplanted for ALL or in ≥CR2 had higher risk of relapse, while those transplanted with active disease had increased RI and NRM. Reduced anti-leukemic effect of allo-SCT with higher relapse rates in ALL in comparison to AML is well known in both non-haplo and haplo-SCT settings [50, 51]. As reported by other groups, outcome of RIC and MAC haplo-SCT in patients transplanted with active disease are poor, with relapse rates above 50 %, increased NRM, and OS below 20 % [13, 17–19, 22], confirming the need for developing innovative transplant strategies for those patients. In this series, patients transplanted in advanced disease phase (CR2 and beyond) had a higher risk of relapse and lower survival rates in comparison to those transplanted in CR1. We also observed a trend in univariate analysis towards reduced LFS and OS after RIC vs. MAC in this subset of patients. These results are in contradiction with those reported in smaller series of patients allo-grafted with RIC or MAC haplo-SCT [13, 22]. However, in their last reports, the Bacigalupo’s group also showed poorer outcome of patients transplanted in ≥CR2 [24, 32]. Our results confirm better transplant outcomes in patients transplanted in CR1 with an OS of about 50 % at 2 years, independently of the intensity of the conditioning and of recipient age (data not shown). Altogether, these results suggest that haplo-SCT should be considered in the first line of treatment in high risk AML and ALL and can be performed with MAC or RIC according to patient’s age and co-morbidities.

Cumulative incidences of acute and chronic GVHD were similar between RIC and MAC. Grade II–IV acute GVHD occurred in 25 % of patients transplanted with RIC and 29 % of those receiving a MAC, while chronic GVHD was observed in 25 and 32 % of them, respectively. These results are in line with the reported incidences of GVHD after T-replete haplo-SCT associated with different strategies of in vivo T cell depletion [13–15, 17–26] and confirm the reduction of GVHD rates with such strategies in a multicenter experience. The use of PB vs. BM stem cells was associated with increased risk of acute and chronic GVHD but had no impact on the incidence of NRM and survivals. While the use of PB is also associated with increased chronic GVHD in other HLA-matched transplant settings [52, 53], its impact on the incidence of acute GVHD is more debatable. In haplo-SCT with RIC and PT-Cy, Castagna et al. could not find significant increase of acute GVHD with PB in comparison to BM [54]. In this study, the use of PB was associated with higher risk of grade II–IV acute GVHD independently of the intensity of the conditioning regimen and of the use of PT-Cy. Because of the heterogeneity of the conditioning and GVHD prophylaxis regimens, we believe that the potential impact of the stem cell source on the incidence of acute GVHD in haplo-SCT needs further investigation.

**Fig. 3:**
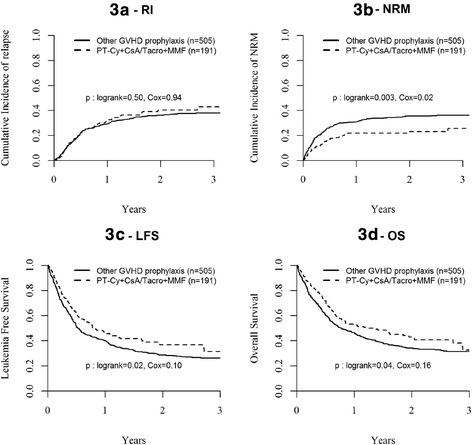
Probability of (*3a*) relapse incidence (RI); (*3b*) non-relapse mortality (NRM); (*3c*) LFS, and (*3d*) OS after haplo-SCT for AL with or without PT-Cy + CsA/Tacro + MMF as GVHD prophylaxis

NRM was similar between RIC and MAC groups. However, NRM was above 30 % in both groups, including in patients transplanted in CR1, which is higher than the levels below 20 % reported with T-replete haplo-SCT independently of the type of GVHD prophylaxis in single centers’ experiences [12, 13, 17–26]. Apart from disease status, the only factor impacting NRM in multivariate analysis was the use of PT-Cy in association to one calcineurin inhibitor and MMF in the prophylaxis of GVHD. NRM with PT-Cy + CsA/Tacro + MMF was closed to 20 % at 2 years (Fig. 3b), as described by the Baltimore’s group [17–19]. Reduction of NRM with PT-Cy was due to decreased mortality from GVHD (data not shown) without significant reduction in the incidence of acute and chronic GVHD, suggesting that PT-Cy ameliorates the severity of GVHD. While there was a trend for an increased incidence of relapse in RIC vs. MAC in multivariate analysis adjusted for disease risk, as observed in non-haplo-identical SCT [38–40], interestingly, patients receiving PT-Cy + CsA/Tacro + MMF did not have increased risk of relapse. Altogether, these data suggest that the association of PT-Cy with one calcineurin inhibitor and MMF represents a safer platform for GVHD prophylaxis in haplo-SCT with reduced NRM without impact on relapse incidence.

In comparison to the data reported by the Baltimore’s group of haplo-SCT performed with a Fludarabine-Cyclophosphamide-low dose TBI RIC and PT-Cy, the data of RIC with PT-Cy in this multicenter experience confirm that this approach is associated with low risks of GVHD and provides lower NRM. Although, PT-Cy is not associated to an increased risk of relapse, its incidence remains however the major post-transplant event with 30 % incidence at 2 years in patients transplanted in CR1 in our series. Thus, although this platform seems safer, strategies for improvement are needed. In this objective, adaptation of conditioning intensity, the use of PBSC together with post-transplant immunomodulation, and preventive anti-leukemia targeted strategies might improve the outcome.

## Conclusions

We recognize that this study has several limitations. First, it is retrospective and registry-based with imperfectly reported cytogenetics data, and the reasons for the choice of the intensity of conditioning regimen and GVHD prophylaxis were unknown but mainly dependent on centers’ protocols and experience. Second, the conditioning regimens and GVHD prophylaxis used were very heterogeneous, and patient characteristics varied among the groups for multiple factors including age, type of leukemia, and disease status at transplantation. Despite these limitations, the results described in this large retrospective study confirm single center experiences of the validity of performing T-replete haplo-CST with MAC or RIC, in particular using PT-Cy as GVHD prophylaxis, in first line treatment of high risk AML and ALL. Prospective comparative studies are required to determine the optimal conditioning and GVHD prophylaxis as well as the place for post-transplant immunomodulation or targeted therapeutic strategies.

## Methods

### Study design and data collection

This was a retrospective multicenter analysis. Data were provided and approved for this study by the acute leukemia working party (ALWP) of the EBMT group registry. The latter is a voluntary working group of more than 500 transplant centers that are required to report all consecutive stem cell transplantations and follow-ups once a year. Audits are routinely performed to determine the accuracy of the data. The study protocol was approved by the institutional review board at each site and complied with country-specific regulatory requirements. The study was conducted in accordance with the Declaration of Helsinki and Good Clinical Practice guidelines. All patients provided written informed consent authorizing the use of their personal information for research purposes. Eligibility criteria for this analysis included adult patients (age >18 years) with AL, transplanted between 2001 and 2012, from an HLA-haplo-identical donor with bone marrow (BM) or G-CSF mobilized peripheral blood (PB) stem cells. All donors were HLA-mismatched at least at two loci (≤8/10) (-A, -B, -C, DRB1, -DQB1). Exclusion criteria were previous allogeneic or cord blood transplantation and ex vivo T cell depleted stem cell graft. Variables collected included recipient and donor characteristics (age, gender, CMV serostatus), disease status at transplant, transplant related-factors including conditioning regimen, pre-transplant immunosuppression (in vivo T cell depletion with anti-thymocyte globuline or alemtuzumab vs. none), stem cells source (BM or PB), GVHD prophylaxis (PT-Cy + CsA/Tacro + MMF vs. others), and outcome variables (acute and chronic GVHD, relapse, NRM, leukemia-free survival (LFS), OS, and causes of death). Regimens were classified as MAC or RIC based on published criteria [55]. Grading of acute and chronic GVHD was performed using established criteria [56]. Chronic GVHD was classified as limited or extensive according to usual criteria [57]. The list of institutions reporting data included in this study is provided in the supplemental data (Additional file 1: Table S1).

### Statistical analysis

The primary end point of the study was overall (OS) and leukemia-free survival (LFS). Secondary endpoints included disease relapse incidence (RI), non-relapse mortality (NRM), engraftment, incidences, and severity of acute and chronic GVHD. LFS was defined as survival without relapse or progression and NRM as death without relapse/progression. The two groups according to the conditioning regimen were compared by the chi-square method for qualitative variables, whereas the Mann-Whitney test was applied for continuous parameters. Univariate comparisons were done using the log-rank test for OS, LFS, and Gray’s test for RI, NRM, and GVHD cumulative incidences. Multivariate analyses were performed using logistic regression for complete remission (CR) rate and Cox proportional hazards model for all other endpoints. Factors differing in terms of distribution between the two groups and factors associated with a *p* value less than 0.15 by univariate analysis were included in the final model. All tests were two-sided. The type I error rate was fixed at 0.05 for the determination of factors associated with time to event outcomes. Statistical analyses were performed with SPSS 22.0 (IBM Corp., Armonk, NY, USA) and R 3.1.1 software packages (R Development Core Team, Vienna, Austria).

## Additional files

Additional file 1: Table S1. List of institutions reporting patients’ data for the study. (DOCX 38 kb)
