# Supplementary material for: Impact of conditioning intensity in T-replete haplo-identical stem cell transplantation for acute leukemia: a report from the acute leukemia working party of the EBMT
Source: J Hematol Oncol. 2016 Mar 15;9:25. doi: 10.1186/s13045-016-0248-3 (PMC4791867; doi:10.1186/s13045-016-0248-3)
Supplement: Additional file 1: Table S1. — List of institutions reporting patients’ data for the study. (DOCX 38 kb) [file 13045_2016_248_MOESM1_ESM.docx]

**Additional file 1: Table S1: List of institutions reporting patients’ data for the study**

| **EBMT centre** | n |
| --- | --- |
| 813 Milano [S Raffaele] | 87 |
| 217 Genova [S Martino] | 58 |
| 756 Rome [Tor Vergata] | 46 |
| 919 Antalya [Medical Park H] | 37 |
| 259 Essen [Univ H] | 32 |
| 440 Kocaeli [Anadolu] | 30 |
| 931 Suzhou [First Soochow] | 26 |
| 248 Pescara [Osp Civile] | 24 |
| 513 Munich [Kl Grosshadern] | 21 |
| 725 St._Petersburg [Pavlov Med Univ] | 19 |
| 401 Hangzhou [Univ H] | 17 |
| 231 Torino [S. Giovanni (CTO)] | 14 |
| 633 Teheran [Shariati] | 13 |
| 587 Reggio_Calabria [Centro Trapianti] | 12 |
| 788 Ancona [Umberto I] | 9 |
| 808 Dresden [Universitaets Kl] | 9 |
| 331 Milano [Ist Europeo Oncologia] | 8 |
| 705 Udine [Univ H] | 8 |
| 712 Würzburg [Medizinische Kl II] | 7 |
| 819 Madrid [H G Marañón] | 7 |
| 205 London [Hammersmith] | 6 |
| 354 Milano [Trapianto Midollo Osseo] | 6 |
| 544 Monza [Osp S Gerardo] | 6 |
| 590 Berlin [Benjamin Franklin] | 6 |
| 763 London [Kings College H] | 6 |
| 204 Ulm [Medizin Kl / Polikl] | 5 |
| 223 Tübingen [UnivTubingen] | 5 |
| 390 Düsseldorf [Heinrich Heine Univ] | 5 |
| 524 Heidelberg [Medizinishce Kl] | 5 |
| 606 Cuneo [S Croce e Carle] | 5 |
| 646 Roeselare [Heilig Hartziekenhuis] | 5 |
| 119 Ascoli_Piceno [Osp Mazzoni] | 4 |
| 163 Piacenza [Osp Civile] | 4 |
| 208 Zürich [208] | 4 |
| 209 Leuven [Univ H] | 4 |
| 287 Rome [S Camillo - Forlanini] | 4 |
|  |  |
| **EBMT centre** | n |
| 387 Birmingham [Queen Elizabeth] | 4 |
| 392 Palermo [Osp V Cervello] | 4 |
| 506 Brugge [AZ Sint-Jan] | 4 |
| 515 Helsinki [Univ Central H] | 4 |
| 625 Nürnberg [Klinikum] | 4 |
| 735 Murcia [H M Meseguer] | 4 |
| 795 Pisa [Az Osp Univ] | 4 |
| 202 Basel [202] | 3 |
| 286 Pavia [S Matteo] | 3 |
| 294 Milano [Osp Niguarda] | 3 |
| 649 Bari [Univ Studi] | 3 |
| 717 Nottingham [City H] | 3 |
| 811 Cagliari [R Binaghi] | 3 |
| 169 Ankara [Gazi Univ] | 2 |
| 215 Brussels [Jules Bordet] | 2 |
| 230 Marseille [Paoli Calmettes] | 2 |
| 258 Jerusalem [Univ Hadassah] | 2 |
| 261 Geneva [261] | 2 |
| 305 Torino [Regina Margherita] | 2 |
| 332 Taranto [Osp Nord] | 2 |
| 346 Sofia [Queen Johanna] | 2 |
| 389 Leipzig [Univ, Haemat/Oncol] | 2 |
| 397 Riyadh [King Faisal] | 2 |
| 526 San_Giovanni_Rotondo [IRCCS] | 2 |
| 534 Cologne [Univ, Medicine] | 2 |
| 561 Thessaloniki [G Papanicolaou G H] | 2 |
| 589 Adana [Baskent Univ] | 2 |
| 614 Hamburg [Univ H] | 2 |
| 617 Ankara [Ibni Sina H] | 2 |
| 640 Ljubljana [Univ Med Ctr] | 2 |
| 658 Bergamo [Ospedale, ematol] | 2 |
| 692 Palermo [La Maddalena] | 2 |
| 718 Pilsen [Charles Univ H] | 2 |
| 726 Liege [University] | 2 |
| 731 Umeå [Univ H] | 2 |
| 754 Tel-Hashomer [Univ Adults] | 2 |
| 789 Avellino [S G Moscati] | 2 |
| 791 Cagliari [Osp A Businco] | 2 |
| **EBMT centre** | n |
| 152 Augsburg [Zentra Kl] | 1 |
| 187 Aydin [Adnan Menderes Univ] | 1 |
| 234 Brussels [St. Luc] | 1 |
| 240 Bologna [S Orsola-Malpighi] | 1 |
| 242 Santander [Valdecilla] | 1 |
| 253 Nantes [Hotel Dieu] | 1 |
| 256 Kiel [UKSH] | 1 |
| 265 Milano [Osp Maggiore] | 1 |
| 266 Uppsala [Univ H] | 1 |
| 271 Innsbruck [Univ H] | 1 |
| 299 Bolzano [Osp S Maurizio] | 1 |
| 302 Zagreb [Univ H Rebro] | 1 |
| 311 Wiesbaden [Kl Diagnostik] | 1 |
| 339 Antwerp [AZ Stuivenberg] | 1 |
| 345 Haifa [Rambam MCH] | 1 |
| 409 Petach-Tikva [Belingon H] | 1 |
| 529 Pesaro [Osp, Transplant Ctr] | 1 |
| 538 Wroclaw [Ctr Cell Transpl] | 1 |
| 557 Pavia [S Matteo] | 1 |
| 558 Munich [Rechts des Isar] | 1 |
| 582 Belgrade [Military Medical Acad] | 1 |
| 598 San_Sebastian [H Aranzazu] | 1 |
| 616 Milano [INT] | 1 |
| 652 Tricase_(Lecce) [C Panico] | 1 |
| 656 Prague [Ist Hematology] | 1 |
| 722 Palma_De_Mallorca [Son Dureta] | 1 |
| 744 Gent [Univ H] | 1 |
| 766 Napoli [Federico II] | 1 |
| 773 Liverpool [Alder Hay] | 1 |
| 775 Paris [St Antoine] | 1 |
| 794 Perugia [Monteluce] | 1 |
| 809 Erlangen [University] | 1 |
| 810 Freiburg [University] | 1 |
| 825 Alessandria [SS Antonio e Biagio] | 1 |
| 862 Ankara [Medicana] | 1 |
| 954 Warsaw [Central] | 1 |
| Total | 696 |
